# Development and Evolution of Dentition Pattern and Tooth Order in the Skates And Rays (Batoidea; Chondrichthyes)

**DOI:** 10.1371/journal.pone.0122553

**Published:** 2015-04-15

**Authors:** Charlie J. Underwood, Zerina Johanson, Monique Welten, Brian Metscher, Liam J. Rasch, Gareth J. Fraser, Moya Meredith Smith

**Affiliations:** 1 Department of Earth and Planetary Sciences, Birkbeck, University of London, Malet Street, London WC1E 7HX, United Kingdom; 2 Department of Earth Sciences, Natural History Museum, Cromwell Road, London, SW7 5BD, United Kingdom; 3 Department of Animal and Plant Sciences, The University of Sheffield, Sheffield S10 2TN, United Kingdom; 4 King's College London, Dental Institute, Craniofacial Development, London SE1 9RT, United Kingdom; 5 Department of Theoretical Biology, University of Vienna, Althanstrasse 14, 1090 Wien, Austria; Team 'Evo-Devo of Vertebrate Dentition', FRANCE

## Abstract

Shark and ray (elasmobranch) dentitions are well known for their multiple generations of teeth, with isolated teeth being common in the fossil record. However, how the diverse dentitions characteristic of elasmobranchs form is still poorly understood. Data on the development and maintenance of the dental patterning in this major vertebrate group will allow comparisons to other morphologically diverse taxa, including the bony fishes, in order to identify shared pattern characters for the vertebrate dentition as a whole. Data is especially lacking from the Batoidea (skates and rays), hence our objective is to compile data on embryonic and adult batoid tooth development contributing to ordering of the dentition, from cleared and stained specimens and micro-CT scans, with 3D rendered models. We selected species (adult and embryonic) spanning phylogenetically significant batoid clades, such that our observations may raise questions about relationships within the batoids, particularly with respect to current molecular-based analyses. We include developmental data from embryos of recent model organisms *Leucoraja erinacea* and *Raja clavata* to evaluate the earliest establishment of the dentition. Characters of the batoid dentition investigated include alternate addition of teeth as offset successional tooth rows (versus single separate files), presence of a symphyseal initiator region (symphyseal tooth present, or absent, but with two parasymphyseal teeth) and a restriction to tooth addition along each jaw reducing the number of tooth families, relative to addition of successor teeth within each family. Our ultimate aim is to understand the shared characters of the batoids, and whether or not these dental characters are shared more broadly within elasmobranchs, by comparing these to dentitions in shark outgroups. These developmental morphological analyses will provide a solid basis to better understand dental evolution in these important vertebrate groups as well as the general plesiomorphic vertebrate dental condition.

## Introduction

Living chondrichthyans include sharks, rays and the holocephalans (chimaeras), which between them fill a diverse suite of marine, and some freshwater, niches. Whilst the sharks are probably best known as high-level predators, this belies the wide diversity of chondrichthyans, which show extremely varied dentitions. Sharks and rays are well known for the presence of multiple generations of teeth forming a ‘conveyor belt’ along the jaw, brought to the functional surface and subsequently lost. However, the development of these dentitions and how teeth are organized into highly functional dentitions characteristic of the group is poorly understood [1]. This lack of understanding makes it difficult to determine dental characteristics of the major chondrichthyan groups and to extrapolate observations from Recent taxa that would allow us to acquire a better understanding of fossil chondrichthyan dentitions. This also hinders comparisons to the development of structural pattern to other major jawed vertebrate (gnathostome) groups such as the Actinopterygii (Osteichthyes), and to the fossil groups ‘Placodermi’ and ‘Acanthodii’. Information from each of these major groups is required before the general characteristics of the vertebrate dentition as a whole can be properly described. In order to better understand the development of chondrichthyan dentitions it is important that the dentitions of sharks, batoids and holocephalans (e.g., [1]) are studied in detail, particularly early in ontogeny. Our focus below is on the Batoidea, comprising a monophyletic clade containing diverse rays, skates, guitarfish and sawfish and representing the most speciose group of extant chondrichthyans.

**Fig 1:**
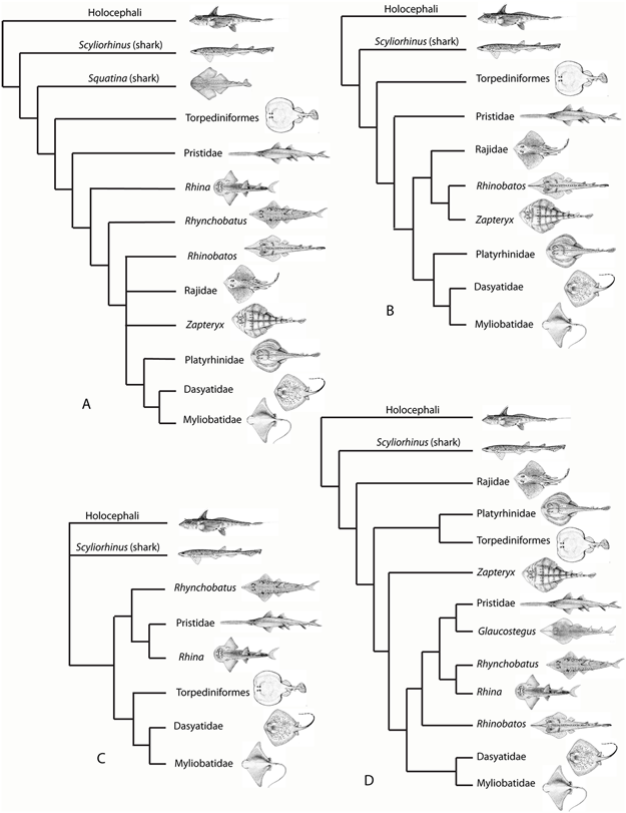
Phylogeny of the Batoidea and selected outgroups. Four selected phylogenies showing the varying topologies and the differing positions of clades such as the Torpediniformes. A. After [10] with batoids as derived sharks. B. After [7] with Torpediniformes in a basal position. C. After [6] with the Torpediniformes as a sister group to the Myliobatiformes, but phylogenetic analysis did not include the Rajidae. D. After [5] with polyphyly of the ‘rhinobatids’ and Torpediniformes as a sister group to the Platyrhinidae. Profile images not to scale, slightly modified from FAO publications under a Creative Commons Attribution-Noncommercial 3.0 Unported License. Species studied are: Rajidae; *Amblyraja doellojurdoi*, *Amblyraja frerichsi*, *Atlantoraja castelnaui*, *Bathyraja griseocauda*, *Bathyraja scaphiops*, *Dipturus batis*, *Dipturus binoculata*, *Dipturus chilensis*, *Leucoraja circularis*, *Leucoraja erinacea*, *Leucoraja naevus*, *Psammobatis normani*, *Psammobatis rudis*, *Raja brachyura*, *Raja clavata*, *Raja microocellata*, *Raja undulata*, *Rioraja agassizi*, *Sympterygia acuta*. Platyrhinidae; *Platyrhinidis triseriata*. Torpediformes: *Discopyge tschudii*, *Narcine* sp., *Torpedo puelcha*. Zapteryx: *Zapteryx brevirostris*. Pristidae: *Anoxypristis cuspidata*, *Pristis perotetti*. Glaucostegus: *Glaucostegus typus*. Rhynchobatus: *Rhynchobatus djiddensis* s.s., *Rhynchobatus* ex. gr. *djiddensis*. Rhina: *Rhina ancylostoma*. Rhinobatos: *Rhinobatos horkelii*, *Trygonorrhina fasciata*. Dasyatidae: *Dasyatis brevis*, *Dasyatis*? *macrophthalma*, *Himantura uarnak*, *Himantura* sp. 1., *Himantura* sp. 2., *Neotrygon kuhlii*, *Pastinachus sephen*, *“Taeniura” lymma*, *Taeniura meyeni*. Myliobatidae and other derived Myliobatiformes: *Aetobatus* ex. gr. *narinari*, *Aetomylaeus maculatus*, *Myliobatis aquila*, *Myliobatis australis*, *Myliobatis californica*, *Myliobatis goodei* s.s., Myliobatis spp., *Rhinoptera javanica*, *Mobula* sp.

The Batoidea contain over half of all extant species of chondrichthyans, with about 630 species spread between up to 23 families. The Batoidea is now considered to form a sister group to all living sharks ([2–8], although some studies previously placed them as derived sharks [9, 10]; Fig. 1A), with these forming the Neoselachii (or Elasmobranchii [11]), a clade that excludes many fossil ‘shark’ groups. While the monophyly of the Batoidea is not in doubt, phylogenetic relationships within the group are uncertain. There has been marked discrepancy in the relative positions of batoid clades in studies based on different phylogenetic methods, with recent molecular analyses either largely based on sharks (e.g., [2, 8]) or lacking members of all major batoid clades [6]. Morphological evidence has been used to suggest Torpediniformes are the most basal batoids (summarized by [10]), as have earlier molecular analyses [12], while more recent molecular analyses resolve the Torpediniformes as more derived than the Rajidae and a sister group to the Platyrhinidae [5] or Myliobatiformes [6]. These batoid phylogenies are summarized in Fig. 1.

**Fig 2:**
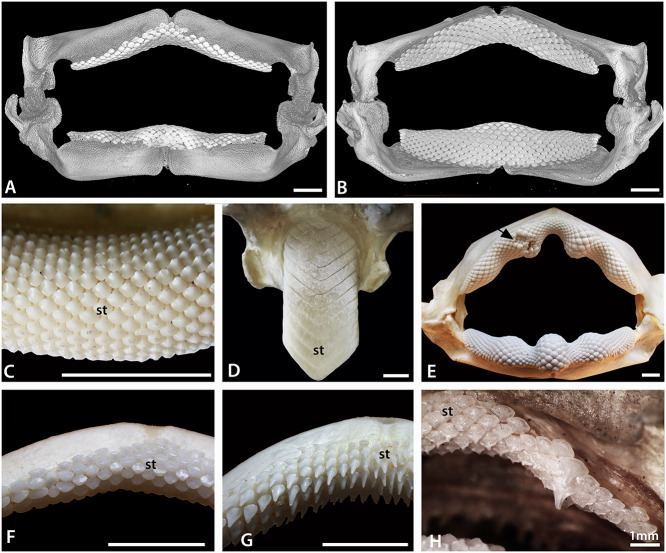
Morphological variation in batoid dentitions. A, B. Labial and lingual views, volume rendered scan of the jaws of an adult female *Raja clavata* (BMNH 2015.1.25.1). C. Symphyseal region of the lower dentition of the ‘rhinobatid’ *Glaucostegus typus* (BMNH 2015.1.25.3), showing alternate row pattern and massive numbers of small teeth. D. Lower dentition of the myliobatid *Aetobatus* ex. gr. *narinari* (BMNH 2015.1.25.4), in which only enlarged symphyseal teeth are present. E. Whole, articulated jaw of *Rhina ancylostoma* (BMNH 2015.1.25.5) showing convoluted pattern of the teeth and a region of malformed teeth (black arrow, see also Fig. 3D in [1]). F. Upper jaw of a female *Raja clavata*, showing homodont dentition of low crowned teeth. G. Upper jaw of a male *Raja clavata* (BMNH 2015.1.25.2), showing a heterodont dentition with tall cusped teeth. H. Upper dentition of a young female of the dasyatid *Neotrygon kuhlii* (BMNH 2015.1.25.6) with enlarged ‘caniniform’ teeth, in S+9 position on the jaw. In this and other figures, symphyseal teeth are labelled ‘st’, the jaw symphysis as ‘S’. All scale bars are 1cm unless marked otherwise.

**Fig 3:**
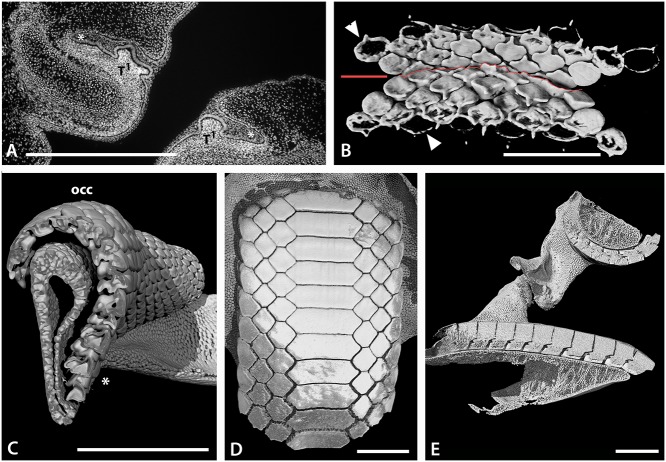
Tooth arrangement in dentition of batoids. A. Histological DAPI (DNA) stained section through upper (left) and lower (right) jaws of an embryonic *Raja clavata*. Teeth (T^1^) developing within the dental lamina (*). B. Volume rendered lingual view (from inside oral cavity) of the developing teeth of an embryo of *Discopyge tschudii* (BMNH 2015.1.25.7) showing progressive mineralization of the teeth on the upper and lower jaws (occlusal surface indicated by red line). C. Volume rendered segment of the lower jaw of a female adult *Raja clavata*, with a vertical section view through tooth files showing migration of teeth from the newest in the dental lamina (*), to oldest beyond the occlusal surface (occ), as well as the close alternate packing of the teeth. D. Volume rendered occlusal view of the lower dentition of an adult *Myliobatis* sp. (BMNH 2015.1.25.10) showing enlarged symphyseal teeth and interlocking of alternate rows of tooth files S+1–3. E. Volume rendered longitudinal (rostral to caudal) section view through both jaws of an adult *Myliobatis* sp. showing close interlocking of the teeth and different degrees of curvature of the occlusal surfaces of upper and lower jaws. Scale bars = 1mm (A, B) or 1cm (C-E).

Despite the constraints of a variably flattened body and enlarged pectoral fins, the Batoidea are morphologically and taxonomically diverse. This high morphological diversity is reflected in their dentitions (e.g. [11, 13–15] and refs therein), with some genera possessing hundreds of minute functional teeth all locked together in a pavement (such as in *Pristis* and *Glaucostegus*; Fig. 2C), while in others the number of teeth in the pavement is reduced to one extremely enlarged symphyseal tooth file (*Aetobatus*; Fig. 2D). Teeth of many taxa of batoids are flat and wide (*Myliobatis* sp.; Fig. 3D), adapted for some degree of durophagy, although more slender and narrow teeth, suitable for predation through grasping active prey, are present in some species. Other taxa, such as members of the Mobulidae (Myliobatiformes), have teeth that are reduced in size and which may be non-functional, at least as far as feeding is concerned, due to a planktivorous diet.

As batoids produce teeth on a continuous basis, much of the information on the arrangement and succession of teeth can be gained from the dentitions of adult individuals. However, initiation of tooth development requires the early stages of dental ontogeny, and for this it is essential to observe teeth of embryonic specimens. In the sharks studied, tooth generation begins at the symphysis, with initiation of the symphyseal tooth proposed as the starting point for timing and placement of teeth in dentition patterning [1]. Our hypothesis is that batoids, as in sharks, possess symphyseal teeth (e.g., Fig. 2, st) initiating the pattern of the dentition in the earliest stages of ontogeny and that this state is primitive and conserved among chondrichthyans. Other dentition patterns to be investigated in batoids, as potentially indicative of the general chondrichthyan condition, include whether the adult number of teeth are present in the earliest ontogenetic stages, and how the pattern unfolds in developmental time (e.g., how do sequential files and rows develop relative to one another), and how aspects of crown and root morphology develop. In this way, we can begin to better understand characteristics of the chondrichthyan dentition and its organization and structural patterning, ultimately for comparison to other gnathostome taxa.

## Materials and Methods

To assess development in the Batoidea, dentitions of a range of modern batoids were studied. Dried and prepared dentitions of over 45 batoid species, including examples of all major clades, were studied (Fig. 1). Additional specimens of adults and late stage embryos of a number of species were selected for more detailed study. Late stage embryos (defined as being from within the mother or egg but having at least some developed teeth) of *Rhinobatos horkelii*, *Myliobatis* sp., *Discopyge tschudii* and *Raja clavata* representing the Rhinobatidae (s.s.), Myliobatidae, Narcinidae (Torpediniformes) and Rajidae (s.s.) respectively (Fig. 1) were studied by x-CT. In addition, neonate specimens (free swimming but with prominent umbilical scars) of *Myliobatis* sp., *Rioraja agassizii* and *Bathyraja* sp. were studied by the same method. Embryos of seven shark taxa were also studied for comparison. It should be noted that the taxonomy of S.W. Atlantic *Myliobatis* is in a state of flux, and the specimens here belong to either *M*. *freminvillii* or an unnamed taxon similar to *M*. *goodei*. All figured specimens are accessioned to the Natural History Museum, London; accession numbers are given in the figure captions the first time each specimen is figured. Specimens of a range of ontogenetic stages of *Raja clavata* and *Leucoraja erinacea* were also cleared and stained for optical study.

Thornback Ray (*Raja clavata*) embryos were kindly donated by the Native Marine Centre, UK (www.nativemarinecentre.com) and reared at the University of Sheffield marine aquarium (14°C), Department of Animal and Plant Sciences, until the required stage of development. Embryos were then removed from their egg cases and lethally anaesthetized in MS-222 (Tricane) before fixation in 4% paraformaldehyde for paraffin sectioning and further analysis. Little Skate (*Leucoraja erinacea*) embryos were kindly donated by Andrew Gillis, Dalhousie University (Canada), and sourced from Woods Hole Marine Biological Laboratories (MA, USA). These early embryos of *L*. *erinacea* were cleared and stained following standard protocols (CS; Alizarin red and Alcian Blue [16]) then dissected and mounted as upper and lower jaws to show sites of first tooth formation. All animals were culled under the Animals (Scientific Procedures) Act 1986 at the University of Sheffield; no manipulation of the animals was performed prior to their culling.

Specimens were studied using x-CT (X-Tek HMX ST CT scanner, Image and Analysis Centre, Natural History Museum; scans volume rendered with VGStudiomax (http://www.volumegraphics.com/en/products/vgstudio-max.html), Avizo (http://www.vsg3d.com/avizo/overview) and Drishti (http://sf.anu.edu.au/Vizlab/drishti); Micro x-CT at Dental Institute, King’s College, London; GE Locus SP, CT Tech scanner with Microview, creating volumes with voxel sizes 6.5μm to reveal structural pattern order of the developing teeth. For *Raja clavata*, we used soft tissue contrast-enhancing stains [17] with 4μm resolution of tooth germs, using X-Radia, with Xray scintillators, and Zeiss optical lenses, in the Department of Theoretical Biology, University of Vienna [17].

In certain figures, images have been combined in Adobe Photoshop to improve clarity; as well teeth have been false colored in Photoshop or Aviso to identify individual tooth rows. Data (including images and rendered files) will be made available at http://chondrichthyes.myspecies.info/.

Terminology used follows [13]. It should be noted that the terminology used for description of chondrichthyan teeth is commonly the same as that used for the teeth of other gnathostomes (such as ‘root’ and ‘cusp’). In the absence of a chondrichthyan-specific terminology, this is standard practice (e.g. [13]) and based on morphological and/or functional similarities and does not imply direct homology.

Nomenclature used to describe jaw position of the teeth has been inconsistent. For instance, in batoids the jaw is often almost straight and perpendicular to the body axis so the terms anterior and posterior are not appropriate. Here, the terms proximal and distal are used to refer to jaw position, where proximal is closest to the neurocranium. As the jaw hinge is closest to the neurocranial attachment for the suspensorium, proximal is taken as a position closest to the hinge between the jaw cartilages, while distal refers to positions closer to the jaw symphysis. Labial and lingual refer to the outside and inside of the jaw, respectively, and occlusal is used to describe the functional jaw surface. Terminology related to dentition patterning follows [1, 11, 18]. The dental lamina is a double, oral epithelium that invaginates deep into the jaw to form successional teeth, organized into developmental timed sets (tooth files, or families), formed from its aboral aspect (away from the oral epithelium) and continuous along the jaws, always within the concavity of the lingual surface of the jaw cartilages [1, 18, 19].

### Batoid dental diversity

**Fig 4:**
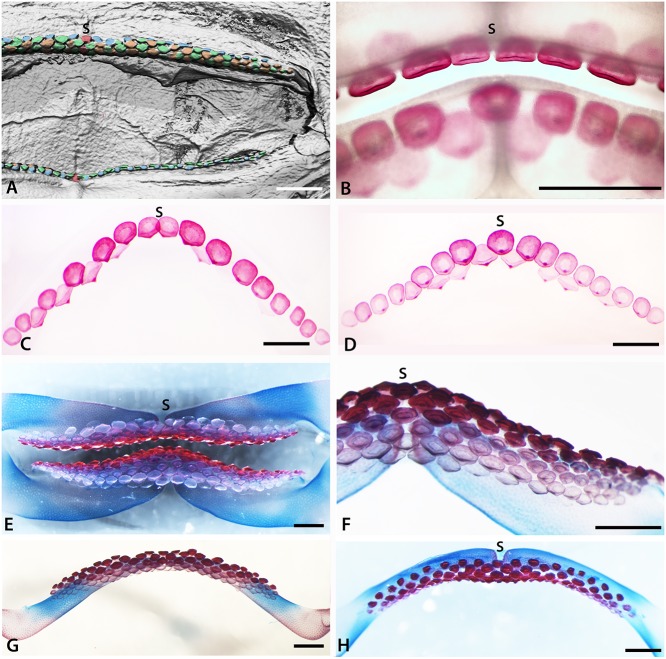
Tooth addition in early ontogeny of rajid and rhinobatid batoids. A. Volume rendered dentition of an embryo of *Rhinobatos horkelli* (BMNH 2015.1.25.13) showing on the upper jaw, the alternate positions of the first formed teeth across the entire width of the jaw. B-D, cleared, Alizarin Red stained preparation of an embryo of *Raja clavata*. The length of the *R*. *clavata* specimen is uncertain sue to it being dissected prior to mounting but is within a late stage of development prior to hatching. The first teeth are well mineralized (Alizarin positive for calcium), relative to the second tooth row (less strong Alizarin Red), in alternate positions. Two parasymphyseal teeth are present next to the jaw symphysis (S in B, C) in the upper first tooth row; in the lower jaw, a symphyseal tooth is present (S in D-F, H) in the first tooth row. B. Labial view of both jaws of the same specimen, showing the relative positions of the first row teeth of upper and lower jaws. C-D. Separated upper and lower jaws respectively. E, F. Cleared and stained lower and upper jaw dentitions of *Leucoraja erinacea*, total length 107mm. F. Portion of the lower dentition of *Leucoraja erinacea* showing alternate rows of teeth with progressive mineralization (degree of Alizarin Red uptake) in the successive tooth rows. G. Lower dentition of *Leucoraja erinacea* in lingual view. H. Labial view of the lower dentition of *Leucoraja erinacea* showing that the first tooth row of 10 positions contains a symphyseal tooth, as in that of *Raja clavata*. Symphysis marked by (S), false colour as in Fig. 6. Scale bars = 1 mm.

**Fig 5:**
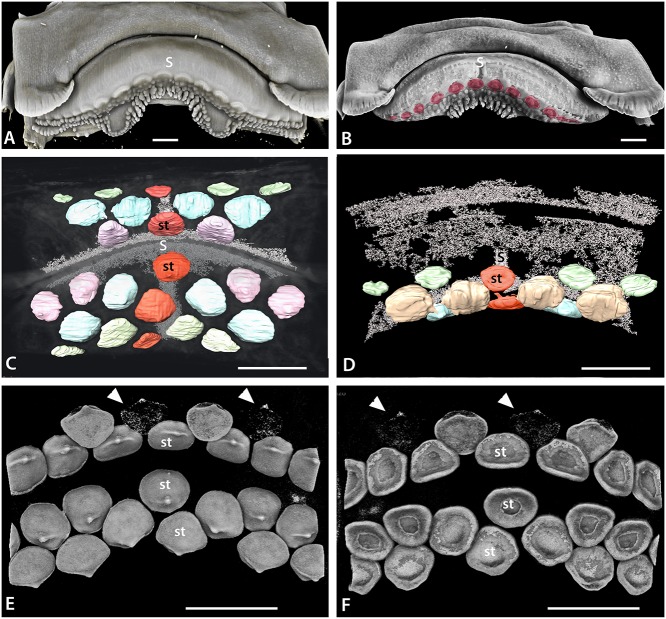
Tooth addition in early ontogeny of *Raja clavata*. A-D. Volume rendered and segmented developing teeth in *Raja clavata* embryos (either VG Studio Max, Drishti, or Avizo). A-B. Embryo 85mm TL, volume rendered upper jaw showing developing teeth as tooth germs under the skin labial to the numerous papillary projections and the symphysis of the palatoquadrate cartilages (S). B. Upper jaw with false colour to highlight the sub-epithelial tooth germs. C-F. Embryo 104mm TL (C, D, stained with iodine and potassium, I2M. Density volume rendered, E, F, unstained). C. Labial view of segmented developing tooth germs in the upper and lower jaws (Avizo); symphyseal tooth (st, red) present in initial row of both jaws, (high density of the connective tissue enhanced at symphyseal junction between cartilages). D. Higher magnification of upper jaw, of lingually rotated jaw to show symphyseal junction (high density tissue) and 3^rd^ row teeth with one at the symphysis (S). E-F. Volume rendered images of the developing teeth (Drishti). Note that the lateral edges of the images are the limits of the render and not the full extent of the teeth. In both jaws there is a symphyseal tooth (st) in the first row and third row of the lower jaw, but the tooth positions may be shifted with growth and not regular. In the upper jaw there is a symphyseal tooth in the first tooth row. Teeth in second row are developing at later times (arrow heads, start of mineralization) relative to the symphyseal tooth Note in E, cusps have a different orientation in 1^st^ and 2^nd^ rows as tooth germs change their developmental positions. F. Lingual view, tooth roots have not started to form and the pulp cavity is open. Scale bars = 500 μm.

Batoids, like sharks, have a polyphyodont dentition, with teeth along the jaw being continuously replaced through life. Each tooth develops on the lingual side of the jaw cartilage within the dental lamina; this differentiates from the oral epithelium (odontogenic band [20–22]) early in embryogenesis. After forming within the dental lamina (Fig. 3A), teeth move labially over the surface of the jaw cartilage to reach the functional (occlusal) position (Figs. 3C, 4B-H and 5C-F) where they contact teeth on the opposing jaw (Figs. 3B, E and 4). Teeth undergo wear during feeding, and continue to move past this functional position, after which teeth are lost. Teeth in each lingual-to-labial file pass through several stages: the development stage (tooth germ), from tooth bud to the whole crown formed; the pre-functional stage where the roots are not fully formed; non-functional when the tooth is not in a jaw position where it will participate in food processing; a functional stage where food processing takes place; and a post-functional stage where the tooth is no longer involved in food processing but has yet to be lost. While most batoids lose teeth soon after the functional stage, in some taxa they may be retained. In the Myliobatidae, for example, sequential and adjacent teeth lock together, through a lingual extension of the tooth base and the close, alternate crown shapes as hexagons form a rigid grinding surface (Fig. 3D, E). In these taxa, lower teeth may be retained for some time, into the post-occlusal stage, forming part of a labially extended tooth plate.

**Fig 6:**
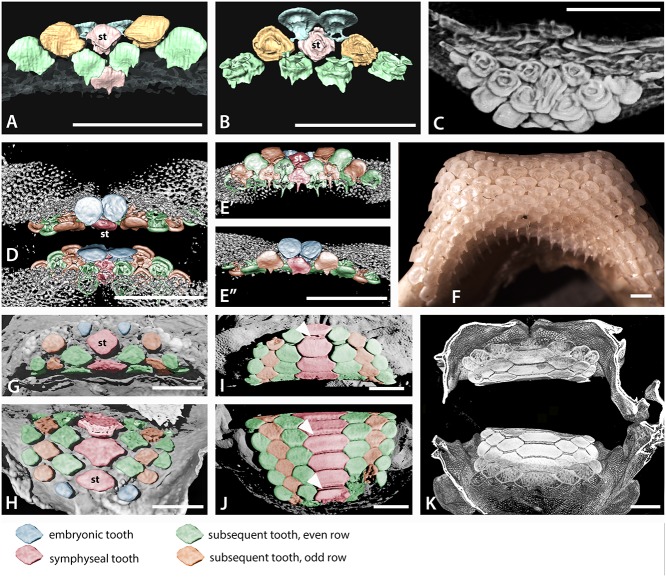
Tooth addition in early ontogeny of batoids. A-E. Volume rendered, segmented, or false colour images of the early dentitions of the torpediform *Discopyge tschudii* (A-C BMNH 2015.1.25.7, D-E BMNH 2015.1.25.8). A. Occlusal surface (labial), with density rendered transparent cartilage, lower jaw, segmented colour for teeth, first and second symphyseal teeth (S, for clarity all teeth in the rows are not shown). However, all tooth rows are shown in same specimen in Fig. 3B, and in false colour images D, E. B. Visceral surface of the same teeth shown in A, to show developing tooth roots. C. Volume rendered view of the upper and lower dentitions of *Discopyge tschudii*, with the lower jaw cartilages digitally dissected to show the bases of the lower teeth. Note the abnormal symphyseal tooth with a labial expansion protruding between the first pair of parasymphyseal teeth. D. Volume rendered upper and lower jaws in labial view, with parasymphyseal pair of teeth (blue) in the apparent first row but subsequent, succeeding row with teeth in alternate positions does have a symphyseal tooth. E’ and E” are from D after rotation of image with lingual and labial views of the lower jaw to show progressive increase in tooth numbers in successive rows, (symphyseal tooth is red). F. Photomacrograph of the lower dentition of an adult *Discopyge tschudii* (BMNH 2015.1.25.9) demonstrating the multitude of tooth files gained by gradual proximal addition of teeth during ontogeny (occlusal view). G, upper, H, lower, jaws of an embryo of *Myliobatis* sp. (BMNH 2015.1.25.11), volume rendered to show occlusal surface of the dentition with the first formed pair of teeth as parasymphyseal (blue) in both jaws; the second row has a larger symphyseal tooth (red) and subsequent teeth in alternating rows of three and four teeth. I, J, volume rendered upper and lower dentition of a neonate of *Myliobatis* sp. (BMNH 2015.1.25.12), occlusal surface showing the fixed number of teeth in each row (S+3) and the gradual enlargement of all through ontogeny (occlusal view). The ‘tongue and groove’ tooth locking is indicated by white arrows. K. Volume rendered jaws of I, J, showing mineralized crowns at occlusal surface compared with younger lingual teeth with less mineralization. False colour coding is used in the dentitions A, B, D, E, G-J as in Fig. 4. All scale bars are 1mm.

Many batoid dentitions show a weak monognathic heterodonty [13], with teeth gradually changing shape between the symphysis and proximal extremities of the jaw. In contrast to monognathic heterodonty, dignathic heterodonty, with different tooth morphologies present on both the upper and lower jaws, is uncommon in most batoids. In taxa where the number of tooth files is very reduced, such as in *Myliobatis*, the symphyseal teeth may be greatly enlarged relative to other teeth resulting in a disjunct heterodonty (Fig. 3D). In some batoid taxa (such as *Rhina* and *Pastinachus*), jaw cartilages have a strongly undulose profile and as a result the shape of teeth varies with both the position relative to high and low points along the jaw and distance between the symphysis and proximal jaw extremities (Fig. 2E). In some other batoids, small ‘caniniform’ teeth are present within the upper jaw (Fig. 2F). In most cases the change in tooth shape along the jaw is still gradual, as a graded tooth size (e.g., Fig. 2E).

Sexually dimorphic dentitions are known within a large proportion of batoids (e.g. [23]), and are probably present in most batoid species. Where sexual dimorphism is present, the teeth of adult males are more cuspate than those of adult females and juveniles (Fig. 2A, B, F, G, H). While this heterodonty may be slight in some taxa, in others, including many species of the Rajidae and Dasyatidae, the differences may be extremely pronounced. In some species there may also be seasonal variation in the degree of heterodonty, with more cuspate teeth developing in males in the breeding season, presumably used for grasping the female during mating [24].

## Results

Batoid dentitions are described from their developmental characters, for the initiation of each tooth that either, shows tooth addition to a new proximal position on the jaw, or the addition of successor teeth in already established loci for each jaw position. These characters are regulated by genes with differential timing for the initiation of new tooth sets proximally and for new successor teeth in jaw positions set up both early, and late in development. Our observations test a proposed model (19) for relative timing of sets ordered by addition from the symphyseal locus, either side in a sequence proximally (laterally), with those of successor teeth proposed to follow this genetically embedded, timed sequence. Developmental characters concerning crown and root development at these developmental times are also described.

### Tooth set initiation and successional addition

#### Early embryonic stages of tooth initiation

Embryonic stages of *Raja clavata* and *Leucoraja erinacea* were used to describe the earliest stages of tooth development. The first tooth germs in *Raja clavata* (prior to mineralization) at the morphogenetic stage are formed from interaction between dental epithelium and condensed mesenchyme (Fig. 3A, ‘T^1^’). Their development starts superficially in the oral epithelium, when a thickened sheet of epithelial cells (odontogenic band) invaginates, forming the dental lamina (Fig. 3A, asterisk). This epithelium is rich in proliferative cells (data not shown here), as observed from PCNA immunohistochemistry of the *R*. *clavata* embryonic dentition at T1-stage (Fig. 3A, T ^1^). The dental lamina continues to proliferate, extending deeply into the mesenchyme of the jaw, restricting provision of the odontogenic cells that initiate continuous tooth addition throughout life to this part of the dental lamina.

Tooth germs of the first tooth row develop along the jaw (*Raja clavata*, Fig. 5A, B), and when these have begun to mineralize (Figs. 4B-D and 5E, F), the next alternate row of tooth germs forms, with lightly mineralized crowns and a lingual cusp that is first to mineralize (Figs. 4C, D and 5E, F, arrowheads). All have flat crowns as in the adult female morphology, with a pulpal opening that closes gradually prior to root development (Fig. 5F); root mineralization occurs well after the formation of the tooth crown, a developmental pattern also seen in all subsequent teeth.

In *Discopyge* once the sixth successive generation of teeth has started to mineralize (seen in crown outline, Fig. 3B, arrow heads), the first teeth have changed their orientation to move into the occlusal position with the upper jaw. These early tooth sets in both jaws are tightly packed, with reciprocal occlusal curves (concave in upper, convex in lower, Fig. 3B). However, these first generations of teeth are probably non-functional (as these embryonic ones are present in embryos confined to the egg case).

Similarly, in *Leucoraja erinacea* (Fig. 4E, F; arrow, st4) the latest full row of successor teeth formed in proximal order, with limited uptake of the Alizarin Red (less calcium), is starting from the symphyseal one, then the next row to form, with alternate teeth, has left and right parasymphyseal teeth first (Fig. 4F, T1-^4^). This Alizarin Red gradation approximates to differential timing of tooth development in each set and along the row, is equated with mineralization levels of xCT density volume renders (Fig. 5E, F arrow heads). In *Leucoraja* the addition of new proximal tooth sets at the jaw margin is probably no longer occurring at this stage (Fig. 4E, F, 15 sets in each half) when approximately the 10^th^ row of successional teeth is forming at the lingual edge.

#### Symphyseal tooth initiation and continuous proximal file addition

The adult dentitions of the torpediniform *Discopyge* are in many respects typical of batoids (Fig. 6F). Both upper and lower jaws show very little heterodonty but contain a large number of small teeth. Embryos of *Discopyge tschudii* have incompletely mineralized jaw cartilages, and as a result the last formed teeth are directly visualized in rendered micro-CT scans (Figs. 3B, arrowheads, 6A-E and 7B). The first tooth row in the embryo of both upper and lower jaws is identified as the most labial row on the jaw; this row comprises a pair of teeth, one either side of the jaw symphysis (Fig. 6A-E, blue teeth); there is no evidence of an initial symphyseal tooth, and the early developmental stage studied suggests that this tooth would be seen if present (i.e., not at a stage where it would have been biologically lost). The parasymphyseal teeth are low and discoidal, with only an incipient cusp, differing from teeth in adults or free-swimming juveniles (Fig. 6A-F). The second tooth row comprises three teeth, a symphyseal tooth (st) and two teeth either side of this, forming files that alternate with those of the first two teeth. At this stage well-differentiated tooth morphology is present, with a small lingual cusp and two root lobes (sensu [13]) with splayed roots separated by a wide groove (Fig. 6B,C). Subsequent tooth rows alternate in a similar pattern to the first two, with rows bearing a symphyseal tooth alternating with those lacking this tooth but having a pair of parasymphyseal teeth. In the third tooth row, a tooth additional to those in previous rows is added proximally each side (further from the symphysis). Each successive tooth row observed has an additional pair of proximal teeth present, so that the number of tooth files along the jaw (added proximally) increases rapidly through the early stages of ontogeny and tooth development. Dentition of adult *Discopyge* is comprised of tooth rows containing a large number of teeth (Fig. 6F), so it is probable that this tooth addition continues throughout life continuously as new proximal sets.

**Fig 7:**
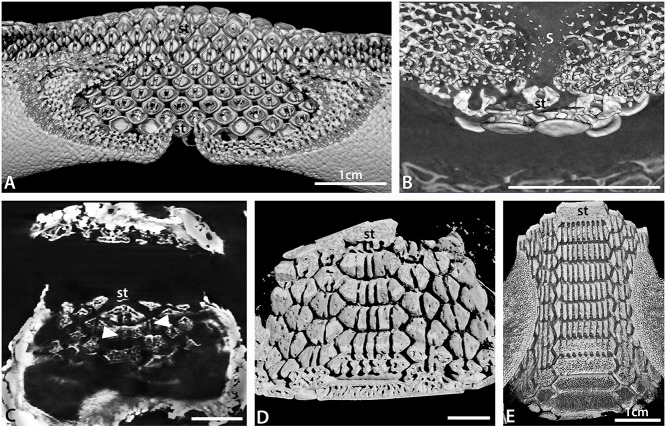
Development of batoid tooth crown size co-ordinated with roots. A. Composite volume render of the basal surface of the lower dentition of a female *Raja clavata* showing the bilobed tooth roots typical of batoids. B. Composite volume render of an embryo of *Discopyge tschudii* where the initial upper teeth show the presence of bilobed roots. C. Render of a basal section through the lower teeth of an embryo of *Myliobatis* sp. showing the presence of one or two, poorly developed grooves within the teeth (white arrows). D. Composite volume render of the basal surface of the lower dentition of a neonate specimen of *Myliobatis* sp. showing up to seven grooves in the roots of the symphyseal teeth and fewer in the other teeth. Many of the grooves are to a greater or lesser extent roofed over. E. Composite volume render of the basal surface of the lower dentition of an adult specimen of *Myliobatis* sp. showing multiple, well developed, grooves in the roots and in the top row their relationship to the crown. All scale bars are 1mm except where indicated.

#### Symphyseal tooth initiation and limited proximal file addition

Whereas adult dentitions of *Discopyge* contain large numbers of small teeth, those of *Myliobatis* differ in having relatively few, very large, teeth even in the adult (Fig. 3D). The symphyseal teeth are especially enlarged, and can comprise over half of the width of the entire dentition. Symphyseal teeth have a large number of root lobes, with the smaller parasymphyseal and proximal teeth also commonly possessing more than two root lobes, particularly in older individuals (Fig. 7D, E). The teeth of embryos of *Myliobatis* are only slightly mineralized, and show little x-ray density contrast relative to the jaw cartilages, as a result they are more difficult to isolate (Figs. 6G, H and 7C).

Tooth development is identical in upper and lower dentitions of *Myliobatis*, with two rudimentary parasymphyseal teeth as the first row (Fig. 6G, H); as with *Discopyge* above, these are the first-developing teeth in the embryo, with a symphyseal tooth being absent. These teeth are oval and very low (comparable to those in the *Discopyge*), and appear to have a flared root considerably wider at the base than the crown (Figs. 6G, H and 7C). The second tooth row comprises three teeth, all of which are considerably larger than those in the first row (Fig. 6G, H). These comprise a symphyseal tooth and a pair of more proximal teeth alternating with the teeth in the first row, identical to those in *Discopyge* (Fig. 6A-E). The two more proximal teeth are rounded, but the symphyseal tooth is somewhat angular and wider than deep, giving an overall ‘diamond’ shaped occlusal profile (st, Fig. 6G, H). The third tooth row lacks a symphyseal tooth and comprises just four tooth files. The teeth are separated and not in contact with each other, and to some extent, angular. No further proximal tooth files are added with successive rows, such that the dentition comprises an alternate series of three teeth, including an enlarged symphyseal tooth, alternating with four teeth. In each successive tooth row, the teeth are relatively larger and more angular, with the symphyseal tooth becoming considerably larger than the other teeth. The roots of these early teeth are very poorly differentiated, and the grooves separating the roots are very shallow. Two grooves (and hence three root lobes) are present on the symphyseal and parasymphyseal teeth, and a single groove separating two roots lobes in more proximal teeth (Fig. 7C).

The dentition of a neonate specimen of the same species of *Myliobatis* shows the transition in tooth morphology from embryo to adult (Fig. 6I-K). The most labial tooth row consists of a symphyseal tooth and one tooth on either side suggesting that the first row of two parasymphseal teeth have been lost, and the general shape and size of the most labial teeth is similar to teeth in the early stages of development in the embryos, including those in the symphysial file. The dentition of this specimen retains the same general pattern seen in the embryo, and also seen in the adult (Fig. 3D). As noted above, rows of three teeth with an enlarged symphyseal tooth alternate with rows of four, not enlarged, but more uniform teeth. All teeth are polygonal and closely packed. In vertical section, the teeth possess the flange and groove system that lock the teeth together in the adult dentition (Fig. 3E). Sequential tooth rows show a marked increase in the width of the dentition, via increase of the width of the symphyseal teeth (see section on root development, below). The mode of tooth mineralization is not clearly evident in the embryonic specimens.

#### Simultaneous symphyseal to proximal tooth initiation addition

In contrast to *Myliobatis* and *Discopyge*, the early dental development in *Rhinobatos* initially occurs across the entire width of the jaw rather than being restricted to the symphysis (Fig. 4A). The first formed teeth are seen only in the upper jaw; in the lower jaw these are concealed behind later teeth due to shrinkage of the specimen before examination. These teeth are extremely small and have not yet moved into a position close to where they would be shed, so any earlier teeth would have been visible if they had been present. The first tooth row extends across the entire width of the lower jaw, clearly alternating with the teeth of the second row. There is a symphyseal tooth at the centre of the first tooth row (red tooth, symphysis, S, Fig. 4A), although slippage of the teeth as the jaw became dehydrated cannot be eliminated. The total number of teeth along each row appears to be similar to the conspecific adult, indicating that there is no subsequent proximal addition of tooth files, again differing from *Myliobatis* and *Discopyge*.

#### Variable tooth initiation pattern

Upper and lower jaws of the two species of Rajidae have a different arrangement of the position of teeth in the initial rows of each although in both, these are followed by a second row, with teeth in alternate positions. In *Leucoraja erinacea* symphyseal teeth are present in the first row of both upper and lower dentitions (Fig. 4E-G), as seen in *Rhinobatos*. In *Raja clavata*, however, there is a difference between the developmental patterns of the upper and lower jaws and between the three specimens examined. In one specimen, the first tooth row in the upper jaw contains two parasymphyseal teeth but no symphyseal tooth, then six to eight teeth either side (Fig. 4B, C). In contrast, the lower jaw has a first row symphyseal tooth that is well developed and separated from the next teeth (Fig. 4B, D). Lingual to these regularly spaced teeth, the second row teeth alternate in positions between those in the initial row.

The other specimens of *Raja clavata* show symphyseal teeth in the first row forming on the upper jaw, as well as the lower jaw, but the arrangement of these teeth relative to the jaw cartilage symphyses is not as clear as in other taxa. One specimen, representing the smallest of the three examined, has an upper symphyseal tooth germ but this is slightly offset relative to the symphysis between the jaw cartilages (Fig. 5A, B). A second, larger, specimen shows developing tooth germs, mineralizing tooth crowns and connective tissue at the symphysis (Fig. 5C, D; soft tissues stained), or mineralizing teeth alone (Fig. 5E, F; unstained). A symphyseal tooth is present in both jaws, associated with the jaw symphyses (S), but both teeth are again slightly offset from the jaw symphysis. Second row teeth on the upper jaw show differing degrees of mineralization in left-right positions relative to the symphyseal tooth (Fig. 5E, F, st). Whilst this dentition initially appears to lack a symphyseal tooth in the first upper row, as the teeth are paired with some in the second row. Closer inspection, however, suggests that the mineralization of the teeth in the second row is irregular, and nor synchronous in adjacent teeth. As a result, it is not possible to use mineralized teeth in the second tooth row to assess tooth position. Additional complexity is seen in the lower dentition of the same specimen, where teeth are extremely close packed and cannot readily be assigned to their correct positions (Fig. 6E-F). Lingual to these regularly spaced teeth, the second row teeth alternate in positions between those in the initial row. In both of the rajid taxa studied, a number of teeth are present in the first formed tooth rows, approximating the number in adults of the same species; with 5–8 teeth in the first half row of *Raja clavata* as opposed to about 15 teeth in each half row in the adult (10–12; Figs. 2A, B, 4E-H and 5A, B). Thus, development of teeth along the row is more rapid than the initiation of subsequent rows of successor teeth (Figs. 4 and 5A, B).

### Changes in crown morphology

In *Discopyge*, the first teeth have a flat, discoidal crown (Fig. 6A-E) over a root with well-developed lobes (Fig. 7B). There is some degree of labial-lingual asymmetry in the crown, with an incipient cusp at the lingual edge (Figs. 3B and 6A, D, E). The initial teeth are closely spaced along the row, and the margins of the teeth are in contact. By the second tooth row, a more adult-like tooth morphology is already established (Fig. 6E, F), with a well-developed cusp and a flared lingual-lateral crown edge. Subsequent teeth have a more elongate cusp and concave labial crown face, but in other respects are similar in shape to the teeth in the second row.

The first teeth of *Raja* and *Leucoraja* are very similar in overall form to those of *Discopyge*, being flat and oval, with a cusp the first to mineralize, but with far less root development (Figs. 4, 5). Incipient cusps are present in the earliest teeth of both genera, in the adult position (lingual), but cusps remain small (Figs. 2G, 4 and 5). This is consistent with the lack of well-formed cusps in the juveniles and mature females of *Raja clavata* (Fig. 2A, B), in contrast to the cuspate teeth of mature males (Fig. 2D, E).

The first teeth in *Myliobatis* (Fig. 6G, H) are similar in overall shape to those in *Discopyge*, although the crown is more robust and the root lobes are not as clearly differentiated (Fig. 7C). In contrast to *Discopyge*, the teeth are widely spaced along the row, being over one tooth diameter apart (Fig. 6H). From that point, the teeth do not start to resemble those of the neonate or adult for several tooth rows (e.g., more proximal teeth remain diamond-shaped rather than hexagonal). The three teeth of the second row show signs of the high degree of heterodonty seen in adult dentitions of *Myliobatis*, with the symphyseal tooth being somewhat larger than the other two; the smaller teeth are also angular in occlusal profile. There is also a groove present on the labial face of the tooth crown, the first indication of the ‘tongue and groove’ tooth locking mechanism seen in *Myliobatis* dentitions (Fig. 6I,J). In the third row, all teeth are somewhat angular, and have a relatively well-developed ridge and groove on the crown edges. Even at this point, however, the teeth are widely spaced. It is therefore evident that the ‘tongue and groove’ tooth locking mechanism appears earlier in ontogeny than the close packing of the teeth that it facilitates.

The first teeth in *Rhinobatos* are small and globular and show little structure (Fig. 4A). Subsequent teeth are not clearly seen, but are wider than deep and have a poorly developed occlusal bulge The tooth morphology is close to that seen in juveniles and adult females of the species; adult males have more strongly cuspate teeth.

### Changes in root morphology

In the majority of batoids, as in sharks, the tooth has a root comprising two root lobes separated by a groove (Fig. 7A, B), which may be closed over in some sharks and some extinct batoids [25]. While teeth of some batoids possess multiple root lobes (e.g., Myliobatidae, discussed below), this remains rare within most clades. Within *Discopyge* the root morphology of the first formed teeth is very similar to that in adult teeth, demonstrating that the template for tooth root morphology must be set within the tooth germ at a very early ontogenetic stage (Fig. 7B). By comparison, the root development in *Raja* and *Leucoraja* is delayed relative to the development of the tooth crown, with no roots observed in the embryonic teeth studied (e.g. Fig. 5E-F). This suggests that whilst the morphology of the roots of fully formed teeth of *Discopyge* and *Raja* are similar, the relative timing of root development is very different.

Within the Myliobatidae and Rhinopteridae multiple root lobes are present on the teeth. Enlarged symphyseal teeth in *Myliobatis* may have up to 30 root lobes with smaller proximal teeth having up to five root lobes; over 50 root lobes may be present on the upper symphyseal teeth of *Aetobatus*. Our observations suggest there is an increase in the number of root lobes through ontogeny in these taxa (Fig. 7C-E). Teeth present in embryos of *Myliobatis* sp. have poorly differentiated roots, with rendered images suggesting a similar histology of the root and interior part of the crown. The base of the root is flat, and grooves are present only as shallow excavations. Despite this, sections through the basal part of the root clearly show that only one or two grooves are present in teeth, and if two are present, one is larger than the other (Fig. 7C). In the teeth of a neonate of the same species, multiple root lobes are present (Fig. 7D). Up to four grooves are present in the roots of symphyseal teeth, two in parasymphyseal teeth and one in other teeth (Fig. 7B). Some of these grooves are partly or almost completely closed over and the spacing across the width of the tooth is irregular. In the adult dentition, up to nine grooves are present in symphyseal teeth, three in parasymphyseal teeth and two in more proximal teeth (Fig. 7E). These are all open and evenly spaced across the tooth. It is therefore evident that the increase of tooth width is directly related to the number of grooves present in the teeth. In addition, the degree of homogeneity of the roots within the teeth increases through ontogeny.

In all *Myliobatis* symphyseal teeth, the relative size and shape of the outermost root lobes remains consistent. In contrast, root grooves within the middle part of the teeth are less regularly distributed (as described in the embryo, above), and addition of new grooves occurs in the central part of the tooth (Fig. 7D, E). Therefore, as the tooth germ, especially of the symphyseal tooth, becomes enlarged during ontogeny, the template for tooth morphology residing in the dental epithelium extends over the root surface and relevant cells divide and map out positions and numbers of the roots as they develop. This would indicate that the enlargement of the tooth germ to form a template for broader teeth must also add multiple roots in the centre of the crown.

## Discussion

Construction of the initial chondrichthyan dentition is highly conserved, especially with the formation of the pattern and the process of lamina-initiated tooth replacement. A better understanding of how the dentition is built through development in chondrichthyan clades such as the Batoidea will allow general characteristics of the structural pattern of the chondrichthyan dentition to be defined. This understanding is crucial before broader comparisons to other major extant groups such as the bony fishes (Osteichthyes) and fossil groups such as the phylogenetically basal ‘Placodermi’ can be undertaken. Recent phylogenetic analyses [26–28] have resolved fossil groups such as the ‘Acanthodii’ as paraphyletic, with some or all ‘acanthodian’ taxa resolved as stem-group chondrichthyans. These new analyses further complicate any assessment of the basal chondrichthyan dentition. Understanding the developmental basis of temporal and spatial order of sequential tooth addition to dentitions within the modern chondrichthyans is, therefore, critical to the interpretation of tooth acquisition and formation of the functional dentition in basal gnathostomes.

### Tooth morphology

In shark dentitions achievement of adult morphology occurs well after initial tooth development [1, 14, 18, 29], and adult tooth shape emerges over many rounds of tooth replacement, a pattern also seen in osteichthyan fishes [28]. However, in the embryonic ray the first sets of teeth (in rows 1 and 2; Figs. 3A, B, 4, 5 and 6) already show a broad flattened morphology, with a single low cusp representing the adult dental morphology (e.g., in *Discopyge*, female *Raja*). This compares with sharks that possess an initial set of teeth that are only lightly mineralized, very small, and with two accessory cusps, ‘simple tooth shards setting up’ the tooth files to start the process that shapes the successor teeth toward the adult phenotype [18]. However, in sharks this ‘shard-like’ mineralized structure is the site of the first cusp (tallest on the crown morphology), also linked to first gene expression with the specific probe for sonic hedgehog, and second expression related to position of lateral cups [18, 19]. Among batoids, *Myliobatis* is more comparable to the shark condition, in that the first teeth differ from those of the adult in terms of size and shape, particularly symphyseally. Root development is also advanced in *Myliobatis* and *Discopyge* (present and developing in the embryo), but delayed in taxa such as *Raja*.

### Dentition development

Within the different batoid taxa studied there are several distinct modes of tooth order addition, involving the symphyseal/parasymphysial teeth as the putative initiators of tooth patterning along the jaw [19], as well as rates of proximal tooth addition along the jaw relative to the addition of successive teeth more lingually.

**Fig 8:**
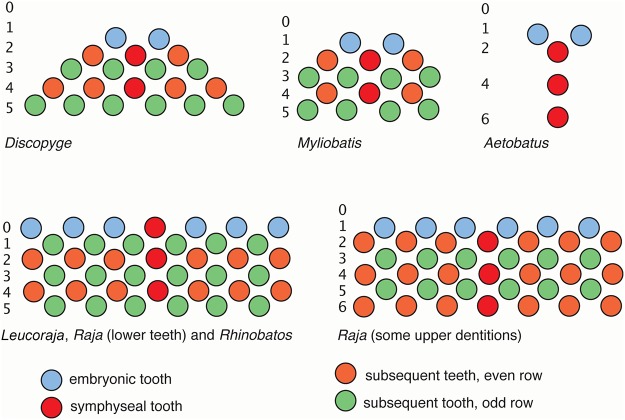
Summary of dental development patterns in the Batoidea. The dentitions of *Discopyge*, *Myliobatis*, *Aetobatus*, *Leucoraja*/*Raja* and *Rhinobatos* are compared, with time order for initiation of tooth rows indicated, proposed as homologous patterns (jaw positions, blue 2,4,6: green 1,3,5. Colour is the same as in Figs. 4 and 6.

The presence of a symphyseal tooth in the first tooth row is variable within the Batoidea. Garman [30] was one of the first to illustrate the presence of two parasymphysial teeth in the first tooth row in embryonic *Rhinoptera* and *Aetobatus* dentition, with a symphyseal tooth first appearing in the second row even though symphyseal teeth dominate the dentition in adults (see Figs. 2D and 8). Our observations have shown that a first row symphyseal tooth is also absent in *Discopyge* and *Myliobatis* as well as in the upper jaw of *Raja clavata*, but present in taxa such as *Rhinobatos*, *Leucoraja* and in the lower jaw of *Raja clavata*. It is evident (from current information) that the presence of a morphological symphyseal tooth in the first row is not fundamental to the development of a batoid dentition. However, as teeth always form initially at, or alongside, the symphysis before bilateral proximal addition, it is probable that focused gene expression at the symphysis is critical to tooth development even if the symphyseal tooth itself is absent. Studies of the tooth development in the shark *Scyliorhinus* have shown that initial tooth development starts at the jaw symphysis, as indicated by the expression of the gene sonic hedgehog [22]. We suggest this symphyseal-driven dentition pattern is shared between sharks + rays (Elasmobranchii). This model will be tested in ongoing molecular developmental studies.

**Fig 9:**
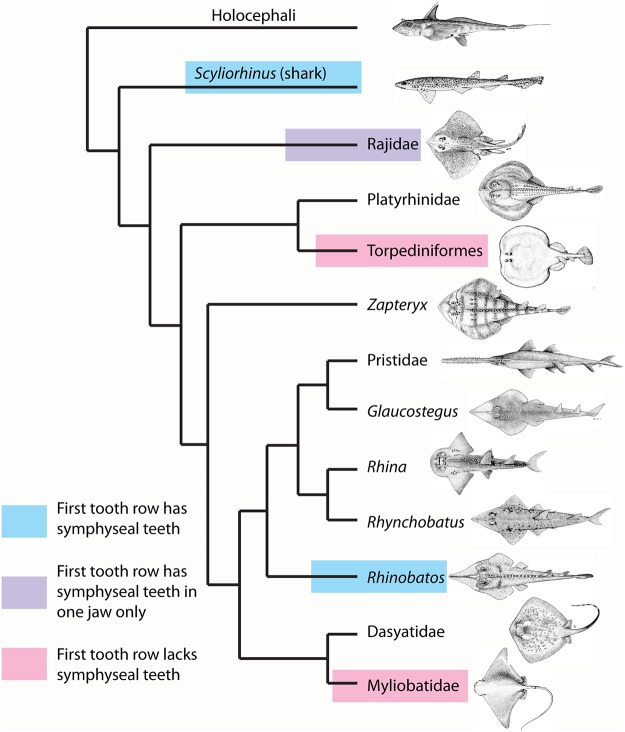
Phylogeny of the Batoidea and selected outgroups with positions of dental development type. Tooth development type as seen in different batoid clades (see Fig. 1E).

In *Scyliorhinus*, consecutive teeth form along the first tooth row away from the symphysis prior to the initiation of the teeth in alternate positions of the second row, so that the first tooth row is complete before the second is initiated [20]. Despite this, tooth initiation in *Scyliorhinus* is not simultaneous along the jaws. Teeth initially form near the symphysis, then in a closely timed developmental sequence from this position, proximally along the jaw. This alternate pattern of tooth addition is seen in all batoids, with the rate of propagation away from the symphysis relative to the rate of development of successive tooth addition (within files) being variable among taxa. The proximal addition of tooth files in *Rhinobatos*, as in *Scyliorhinus*, is more rapid than those added as successive teeth, i.e. before the alternate rows are initiated. In the Rajidae the proximal propagation is still rapid, but somewhat slower than development of successive tooth rows, so that the initial tooth row contains less than the full complement of teeth (Fig. 5 versus Fig. 2A, B). In *Discopyge* and *Myliobatis*, the rate of proximal addition is very slow relative to the generation of successive tooth rows, and as a result only one additional tooth is added proximally each side (Fig. 6). It is therefore evident (see Fig. 9) that slowing of the positional tooth addition in *Discopyge* and *Myliobatis* is convergently derived. There does appear to be a general reduction of proximal tooth addition in the group as a whole, while maintaining the alternate pattern of row addition (proximo-distal) characteristic of the Neoselachii [1].

## Conclusions

Our observations on embryonic and post-embryonic dentitions of the chondrichthyan group Batoidea suggest that despite substantial diversity there are shared batoid characters requiring further comparison to outgroup taxa (e.g., sharks), including the presence of an alternate dentition. Further evidence regarding genetic regulation of a symphyseal region in patterning the initial and subsequent tooth rows in the Batoidea will depend on pending molecular developmental data. Similarly, to determine relative rates of proximal tooth addition versus addition of successive tooth rows will require complete series of developmental data from all significant groups. These comparisons will allow us to establish the phylogentically basal condition for elasmobranchs. Similarities to the shark dentition (e.g., early presence of initiator symphysial region) indicate shared characters for the Elasmobranchii as a whole. Some of these characters are more problematic, for example, whether the alternate dentition characteristic for the Batoidea is plesiomorphic or derived for Elasmobranchii is still to be determined [1] and requires data on selected chondrichthyan taxa showing a dentition composed of single tooth files (e.g., *Squatina*, *Chlamydoselache* [31]). Determining other shared elasmobranch characters, however, will depend on a stable morphological/molecular phylogeny to determine the basal batoid condition. This impacts characters such as rates of proximal tooth addition, being notably reduced in *Discopyge* (Torpediformes), currently basal batoids in morphology-based phylogenetic analyses [9, 10], but more derived in molecular analyses (Fig. 1 [5, 6]). Interestingly, Torpediformes + Myliobatidae is recovered in some of these analyses (Fig. 1C [6]), and would be characterized by this reduction in proximal tooth addition.

Further, we will be able to test how these characters may be controlled in development, for example, with genetic factors that might restrict later timed tooth families along the jaw and initial activator/inhibitor patterning gene interactions (i.e. Hedgehog, Ectodysplasin and Wnt signaling molecules) emerging from the first embryonic morphogenetic stages (odontogenic band) that would allow symphyseal tooth sites to expand and create one large-sized tooth to achieve specialized dentitions at the extremes of morphological diversity (i.e. *Myliobatis*, *Aetobatus*). Because at least three chondrichthyans are now considered ‘model’ organisms for developmental studies (*Scyliorhinus canicula*, *Raja clavata*, *Leucoraja erinacea*), it is now possible to study the evolution and development of dental and general diversity in these chondrichthyan clades that have generated extreme food processing modules, including crushing pavement dentitions, to a more gripping/tearing dentition.

The hypothesis that a symphyseal-driven dentition pattern is shared between sharks + rays (Elasmobranchii) is fully supported by data given here, and is a distinction between? Chondrichthyes and Osteichthyes, the latter have a different initiation site for each dentate bone [19]. We intend to test this hypothesis with data on the genetic regulatory basis of the dentition pattern in forthcoming papers. Ultimately it will allow the general vertebrate condition for a dentition at the origins of jaws to be proposed, and the fossil types in stem vertebrates, assessed and analysed for these characters.
